# Age and gender differences in misperceptions of body shape in a Taiwanese population

**DOI:** 10.1186/s40337-023-00837-5

**Published:** 2023-07-03

**Authors:** Hui-Ching Weng, Sheng-Mao Chang, Jason C. Hsu, Yung-Ning Yang, Chung-Ying Lin

**Affiliations:** 1 Institute of Gerontology, College of Medicine, National Cheng Kung University, Tainan City, Taiwan; 2 Institute of Allied Health Sciences, College of Medicine, National Cheng Kung University, 1 University Road, East Dist., Tainan City, 701401 Taiwan; 3 Department of Statistics, National Taipei University, New Taipei City, Taiwan; 4 International PhD Program in Biotech and Healthcare Management, College of Management, Taipei Medical University, Taipei City, Taiwan; 5 Research Center of Health Care Industry Data Science, College of Management, Taipei Medical University, Taipei City, Taiwan; 6 Clinical Big Data Research Center, Taipei Medical University Hospital, Taipei Medical University, Taipei City, Taiwan; 7 Department of Pediatrics, E-DA Hospital, I-Shou University, Kaohsiung, Taiwan; 8 School of Medicine, I-Shou University, 1, Sec. 1, Syuecheng Rd., Dashu District, Kaohsiung, 84001 Taiwan; 9 Biostatistics Consulting Center, National Cheng Kung University Hospital, College of Medicine, National Cheng Kung University, Tainan City, Taiwan; 10 Department of Occupational Therapy, College of Medicine, National Cheng Kung University, Tainan City, Taiwan; 11 Department of Public Health, College of Medicine, National Cheng Kung University, Tainan City, Taiwan

**Keywords:** Age difference, Gender differences, Misperceptions of body shape, The Taiwanese population

## Abstract

**Objectives:**

Most studies of body size perception have been performed in adolescents, and most focus on gender differences in accurate perception of body size. This study investigated misperceptions of body sizes among males and females at different stages of adulthood in Taiwan.

**Designs:**

In-person home interviews were used to proportionally and randomly select 2095 adult men and women to answer the East Asian Social Survey. Participants were divided into 18–39, 40–64, and 65 + age groups. The main variables analyzed were self-perceived body size and standardized BMI.

**Results:**

Women, unlike men, were more likely to misperceive their body size as being overweight (OR = 2.92; *p *< .001). People with higher self-perceived social status were less likely to misperceive themselves as overweight (OR = 0.91; *p *= .01). People with college educations were 2.35 times more likely to overestimate their body size as being heavier than they were (*p *< .001) and less likely to underestimate it as being thinner than they were (OR = 0.45; *p *< .001). Women 18–35 and 36–64 years old were 6.96 and 4.31 times more likely (*p *< .001) to misperceive themselves as being overweight than women 65 or older, who were more likely to misperceive themselves as being too thin. There were no significant differences in body size misperceptions among the three age groups of adult men (*p *> .05). We found no different significant discrepancies between self-perceived body size and actual BMI between the older men and women (*p *= .16). However, younger and middle-aged men were 6.67 and 3.1 times more likely to misperceive themselves as being too thin than women in their same age groups (OR = 0.15 and OR = 0.32, respectively).

**Conclusions:**

Age and gender affect self-perceptions of body size in Taiwan. Overall, women are more likely than men to misperceive themselves as being too big, and men are more likely than women to misperceive themselves as too thin. Older women, however, were more likely to misperceive themselves as being too thin. Clinicians and health educators should know that people’s perceptions and concerns regarding their body size vary by age and gender.

**Supplementary Information:**

The online version contains supplementary material available at 10.1186/s40337-023-00837-5.

## Introduction

Weight control has become a well-known multi-million-dollar industry as people seek to maintain standards of beauty and improve their health. A significant portion of the global population has reported dieting, with 77% in the US Pound-of-Prevention Database and 86% in the Weight-to-Be Database [1]. Worldwide, 42% of adults reported trying to lose weight, with a higher prevalence in Europe and Central Asia (61.3%) [2]. In Asia, 45.6% of Korean girls and 32.3% of boys had attempted weight-related diets [3], while in Sweden, 69% of women and 59% of men desired weight loss [4]. Self-perceptions of body size significantly influence dieting behavior, and both overestimation and underestimation can negatively impact weight control and quality of life [5–8]. Overestimation in 20% of women and 5% of men in Europe led to unnecessary dieting, meal skipping, and over-exercise [9]. Underestimation of body size is also problematic, as it is associated with more attention-seeking behavior and internalized distress, such as anxiety, depression, social withdrawal, and somatic complaints [10]. One U.S. study identified that women were significantly more likely than men to overestimate their body size (77.3% vs. 47.8%). Similarly, Whites (64.8%) significantly exceeded Blacks (46.3%) and Hispanics (45.6%) in body size overestimation [11]. The odds of self-perceived overweight were also notably higher among individuals with a higher income (OR = 1.5) and education (OR = 1.6) [7, 11]. In Korea, men underestimated their body size more than women (40.35% vs. 25.2%) [12]. Factors such as younger age, male gender, nonwhite ethnicity, low income, and recent measurement contributed to misperception of body weight among obese participants, with odds ratios (ORs) ranging from 2.9 to 7.9 (all *p *< 0.01) [13].

Examining data from various countries, it's clear that weight status misperceptions, including both overestimation and underestimation, significantly differ across genders. For instance, in the United States, most women self-perceive as being overweight (62.3%), while most men perceive themselves as being about the right weight (48.9%) [14]. However, the accurate rates of body size show a different picture, with 44.8% of women and 40.1% of men being overweight, while only 1.6% of women and 0.8% of men are underweight [14]. Another US study indicates that, in the US adult population, accurate perceptions of body type are 59.4%, 68.3%, and 61.9% for underweight, about the right weight, and overweight, respectively [7]. The picture is different in other countries. In Australia, the rates for accurate perception of weight status are 31.0%, 85.9%, and 65.7% for underweight, healthy weight, and overweight individuals, respectively. However, there's a significant rate of underweight misperception among 32% of men and 23% of women and overweight misperception among 5% of men and 14% of women [15]. In Korea, the rates for accurate weight perception are slightly lower than in Australia, with 60.7% of men and 56.8% of women accurately perceiving their weight status. Furthermore, misperceptions of underweight and overweight are noted among 27.2% and 12.2% of men, and 15.6% and 27.6% of women, respectively [16]. In Nigeria, accurate perceptions of body type are considerably lower, with 15.9%, 53.7%, and 33.3% for underweight, about the right weight, and overweight, respectively [17]. Generally, men show higher underweight misperceptions, while women have higher overweight misperceptions.

Nevertheless, there remains a lack of research attention paid to the possible effect of the different adult age ranges on weight perception, and this lack of research data might obscure age-related differences in adults. One study by Chang and Christakis [14], has investigated both gender and age differences in weight perception. That study found that younger (20 to 34 years) and middle-aged (35 to 54 years) females were more likely to overestimate their body size than older females (> = 55 years) (OR = 3.42; 95% CI 2.72–4.27 and OR = 2.59; 95% CI 2.17–3.10, respectively). This age difference was also found among male adults, though less significant (OR = 1.41; 95% CI 1.17–1.70 and OR = 1.52; 95% CI 1.25–1.84–4.27, respectively). Bouzas and Bibiloni [5] conducted a systematic review of 57 studies specifically focusing on adults aged 55 years and above. The review revealed women generally perceive themselves as overweight more often than men and are more likely to take action to control their weight. They tend to have higher expectations for weight loss and often perceive their bodies as more prominent than they are. In terms of attractiveness, women believe men prefer thinner silhouettes than men do, indicating a discrepancy in body image expectations between genders. Moreover, the study revealed that satisfaction levels concerning body weight differ among people from various ethnic backgrounds. It also suggested that as individuals grow older, their body shape acceptance increases, and their weight expectations lessen. This implies that with age, people's priorities shift towards health, interpersonal relationships, and familial ties, moving away from the societal ideals of physical attractiveness.

In summary, although most of the weight or body size perception studies have investigated the association of gender and race with perceptions of body size, none except for Chang and Christakis [14] have studied the association between adult age ranges and body size perception in males and females. And no such study has been performed on adults in East Asia. Though it may seem too obvious to mention, both gender and age are important factors when designing weight control programs. In this study, we utilized data from the 2012 East Asian Social Survey (EASS) for Taiwan to investigate factors affecting diverse body perceptions across age groups and then examined factors contributing to accurate perceptions or misperceptions of body size.

## Methods

This cross-sectional research utilized data gathered from the East Asian Social Survey (EASS), which can be accessed online [18]. The EASS, launched in 2003, is a biennial social survey administered in four East Asian areas (Mainland China, Japan, South Korea, and Taiwan). It was established to encourage comparative studies of diverse aspects of social lives in East Asia. Topics vary from year to year, and sometimes from area to area. Utilizing open-source, anonymized survey data, our study was exempt from ethical review. With no identifiable links to respondents, the study inherently preserved privacy, mitigating harm or distress risks. Additionally, the research didn't involve sensitive topics or vulnerable groups. Thus, given these factors, the Human Research Ethics Committee at National Cheng Kung University deemed an ethical review unnecessary. In 2012, the survey topic administered to Taiwan and South Korea was health. In-person home interviews were used to proportionally and randomly select 2,095 adult men and women to answer the East Asian Social Survey. The study used stratified multistage probability sampling based on eight demographic and ecological variables. These variables classified 358 Taiwanese townships and cities into seven levels. After geographic adjustments, they were combined into 19 strata. Following McKenzie and Directorate [19], to avoid nonresponse bias or invalid responses, the total sample size was increased and adjusted according to the response rates in previous national large-scale studies. We observed an increase in sampling coefficients, ranging from 1.4 to 2.6 times the sample size of the pilot study (n = 150) and from 1.4 to 3 times for the subsequent primary investigation (n = 2000) [20].

Survey items included perceived social status, self-reported BMI, self-perceived body size. Self-perceived status was measured using the MacArthur Scale of Subjective Social Status, a single-item measure that assesses a person’s perceived rank relative to others in their group [21]. It was measured using the question: “In our society, some groups tend to be positioned toward the top and others toward the bottom. Where would you currently put yourself on the scale (1 = bottom; 10 = top)? BMI was calculated as weight in kilograms divided by height in meters squared. In order to compare our findings with Western countries, we used the following cutoff points in BMI to categorize the participants into three BMI groups: underweight (BMI < 18.5), normal weight (18.5 <  = BMI <  = 24.9), and overweight (BMI >  = 25.0).

Many studies used one question to assess "weight perception," such as can be found in studies conducted in the Netherlands [10], England [22], and South Korea [23], with Likert scales ranging from three to five. The choice to use a single question for assessing perceived body size can be attributed to its simplicity, cost-effectiveness, and ease of implementation. Our study assessed weight perception by the question, "How do you view your present body size?" The responses to this question ranged from one to five: (1) underweight, (2) slightly underweight, (3) neither underweight nor overweight, (4) slightly overweight, and (5) overweight. Responses 1 and 2 were considered perceptions of being underweight, response three was considered a perception of being the right size, and responses 4 and 5 were perceptions of being overweight.

Concordance between actual weight and perceived body size was calculated based on the deviance of perceived body size from the actual BMI category. To compare our findings with those of others, we used the WHO BMI cutoff points to categorize subjects into three BMI groups. Deviation from 0 indicated the extent of body size misperception, as described by Quick, Nansel [24]. Scores indicating that respondents perceived their body size to be smaller than their actual BMIs were defined as "misperceived underestimates," scores indicating that respondents perceived their body size to be larger than their actual BMIs were defined as "misperceived overestimates," and scores indicating that respondents' perceived body size matched their actual BMIs were defined as "accurate perceptions of body size." Ages were divided into three distinct categories: 18–35, 36–64, and 65 + years old. These divisions represent varying stages of human development and the associated societal roles [25]. For instance, the majority of Taiwanese young adults aged 18–35, around 80% in 2021, were engaged in higher education, focusing on their academics and career beginnings [26]. Moreover, we also consider public policy and legal aspects. These considerations encompass factors such as age limits, including the official retirement age set at 65, which determine legal adulthood and qualification for social security benefits.

Continuous variables were expressed as mean, standard error, and minimum and maximum. Categorical variables were expressed in percentages of all possible categories. Multinomial logistic regressions were used to analyze the relation between different weight perceptions (i.e., overestimated, underestimated, or accurately estimated body size) [27] and independent variables, including age (18–35 years; 36–64 years; and 65 + years), gender, education, job status, social status, housing income, health status, marital status, and some of their interactions.

Moreover, the reference groups in independent variables were male for gender, 65 + for age, and elementary school and below for education and perceived low social status for social status. We identified three categories from the variables "Concordance between measured weight and self-weight perception": underestimation inaccuracies, accurate body size awareness, and overestimation inaccuracies. Although most classical logistic regression models are based on binary responses, we had three perceived body size groups. Therefore, nominal logistic regression was used for this study with the accurately estimated body size groups serving as a reference. Several independent variables were dropped by backward elimination. Odds ratios were reported with 95% CI induced from the Wald test. The Wald test employed is the square-root variant of the Wald chi-square test, specifically applied to evaluating a singular parameter. All statistical operations were performed using the *net* package available under the R language [28].

## Results

As can be seen in Table 1, a summary of subject characteristics analyzed by three age groups (18–35, 36–64, and 65 +), those between 36 and 64 had the highest actual BMIs (mean = 24.26), followed by those 65 years old and above (mean = 23.94), and then those 18 to 35 (mean = 22.83) (*p *< 0.001). The oldest group had the lowest perceived household income (*p *< 0.001). No group differences were observed in perceived social status (*p *= 0.225). One hundreds of nineteen (5.7%) participants were labeled as "underweight" (BMI ≤ 18.49), 1281 (61.1%) were designated as "normal weight" (BMI 18.5–24.99), and 695 (33.2%) individuals were categorized as overweight (BMI ≥ 25). Almost sixty-five percent of those between 18- and 35 years old, 58% of those between 36 and 64 years old, and 60.9% of those 65 years or older had normal weights (BMI 18.5–24.99) (*p *< 0.001). Approximately 52.5% of the younger group, 53.2% of the middle-aged group, and 31.0% of the older group considered themselves overweight, while 16.2%, 10.8%, and 14.8% of these respective groups perceived themselves as underweight (*p *< 0.001). Self-perceptions are not always accurate. For participants in the younger, middle-aged, and older age groups, 31.5%, 20.7%, and 11.1% respectively overestimated their body size, while 10.5%, 16%, and 25.2% underestimated it (*p *< 0.001).

**Table 1:** Characteristics of subjects by age group

Variable	Age groups								*p*
	All		18–35		36–64		≥ 65		
	(n = 2199)		(n = 838)		(n = 977)		(n = 384)		
	Mean	SD	Mean	SD	Mean	SD	Mean	SD	
BMI (n = 2095)	23.65	3.77	22.83	4.21	24.26	3.38	23.94	3.26	< 0.001
Perceived household income (1–5)	2.74	0.70	2.84	0.63	2.73	0.72	2.55	0.76	< 0.001
Perceived social status (1–10)	4.95	1.57	5.02	1.46	4.91	1.61	4.88	1.69	0.225

	n	%	n	%	n	%	n	%	*p*
Gender (female)	1113	50.61	402	47.97	513	52.5	198	51.6	0.144
Marital status (married)	1299	59.1	256	30.5	790	80.9	253	65.9	< 0.001
Job (with job)	1370	62.3	642	76.6	667	68.3	61	15.9	< 0.001
Educational level									
Primary school and below	475	21.6	2	0.2	222	22.7	251	65.4	< 0.001
High school	816	37.1	247	29.5	482	49.3	87	22.7	< 0.001
College and above	908	41.3	589	70.3	273	27.9	46	12.0	< 0.001
BMI group									
BMI ≤ 18.49 (underweight)	119	5.7	87	10.6	21	2.2	11	3.4	< 0.001
BMI 18.5–24.99 (normal weight)	1281	61.1	533	64.8	550	58.0	198	60.9	
BMI ≥ 25 (overweight)	695	33.2	202	24.6	377	39.8	116	35.7	
Weight perception									
Underweight	299	13.6	136	16.2	106	10.8	57	14.8	< 0.001
Normal	822	37.4	263	31.4	351	35.9	208	54.2	
Overweight	1078	49.0	439	52.4	520	53.2	119	31.0	
Concordance between measured weight and self-weight perception									
Misperceived underweight	320	15.3	86	10.5	152	16.0	82	25.2	< 0.001
Rightly perceived weight	1284	61.3	477	58.0	600	63.3	207	63.7	
Misperceived overweight	491	23.4	259	31.5	196	20.7	36	11.1	

As can be seen in Table 2, women were 2.92 times more likely than men to overestimate their body size (95% CI 1.36–6.26; *p *= 0.01). People with higher educational levels (high school, OR = 1.64; college and above, OR = 2.35) were more likely to overestimate their body size, compared to those with elementary school educations or below. At the same time, individuals with high school (OR = 0.72; 95% CI 0.50–1.03; *p *= 0.07) or college-level (OR = 0.54; 95% CI 0.36–0.82; *p *= 0.01) education were less likely to underestimate their body size compared to those with elementary education or below. This study found an interaction effect between sex and age and their effect on weight perception. Therefore, we reorganized Table 2 to create Table 3 to include the main effects, interaction effects, and total effects. Table 3 reveals that men's weight misperceptions did not differ by age, whether underweight or overweight. However, women demonstrated varied likelihoods of misperceiving their body size across different age groups. In all age groups, women were more likely to perceive themselves as overweight than men of the same age (young: OR = 6.96, *p *< 0.001; middle-aged: OR = 4.31, *p *< 0.001; old: OR = 2.92, *p *= 0.01, respectively). Younger, middle-aged, and older women were approximately seven times, four times, and three times more likely, respectively, to misperceive themselves as overweight compared to men of the same age group.

**Table 2:** Nominal Logistic regression models analyzing select subject characteristics by misperceived overweight and misperceived underweight

Variables	Coefficient ($\widehat{\upbeta }$)	S.E	*p*	OR	95% CI	
					Lower	Upper
*Misperceived overweight*						
Intercept	− 2.17	0.38	< 0.001	0.11	0.05	0.24
Young age	0.37	0.34	0.28	1.45	0.74	2.84
Middle age	0.24	0.35	0.49	1.27	0.64	2.49
Gender (male = ref)	1.07	0.39	0.01	2.92	1.36	6.26
Educational level (elementary school or below = ref)						
High school	0.49	0.20	0.01	1.64	1.11	2.42
College or above	0.85	0.21	< 0.001	2.35	1.55	3.57
Perceived social status (1–10)	− 0.09	0.04	0.01	0.91	0.84	0.98
Middle age*female	0.15	0.43	0.72	1.16	0.50	2.69
Young age*female	0.50	0.43	0.24	1.64	0.71	3.78
*Misperceived underweight*						
Intercept	− 0.51	0.27	0.06	0.60	0.35	1.03
Young age	− 0.21	0.24	0.39	0.81	0.51	1.30
Middle age	− 0.02	0.21	0.92	0.98	0.64	1.49
Gender (male = ref)	− 0.38	0.27	0.16	0.68	0.40	1.16
Educational level (elementary school or below = ref)						
High school	− 0.33	0.18	0.07	0.72	0.50	1.03
College or above	− 0.61	0.21	< 0.001	0.54	0.36	0.82
Perceived social status (1–10)	− 0.02	0.04	0.64	0.98	0.91	1.06
Middle age*female	− 0.75	0.34	0.03	0.47	0.25	0.91
Young age*female	− 1.34	0.45	< 0.001	0.26	0.11	0.64

*ref* Reference group

**Table 3:** Main effects, interaction effects and total effect of gender and age on misperception overweight and underweight

Main effects of gender		Main effects of age		Interaction effects	Total effects			
	Coefficient ($\widehat{\upbeta }$)		Coefficient ($\widehat{\upbeta }$)	Coefficient ($\widehat{\upbeta }$)	Coefficient ($\widehat{\upbeta }$)	s.e	*p*	OR
*Misperceived overweight*								
Female	1.07	Young	0.37	0.5	1.94	0.33	< 0.001	6.96
		Middle	0.24	0.15	1.46	0.32	< 0.001	4.31
		Old (ref)			1.07	0.39	0.01	2.92
Male (ref)		Young	0.37		0.37	0.34	0.28	1.45
		Middle	0.24		0.24	0.35	0.49	1.27
		Old (ref)						
*Misperceived underweight*								
Female	− 0.38	Young	− 0.21	− 1.34	− 1.93	0.4	< 0.001	0.15
		Middle	− 0.02	− 0.75	− 1.15	0.24	< 0.001	0.32
		Old			− 0.38	0.27	0.16	0.68
Male (ref)		Young	− 0.21		− 0.21	0.24	0.39	0.81
		Middle	− 0.02		− 0.02	0.21	0.92	0.98
		Old (ref)						

Total effects are the sum of Coefficient ($\widehat{\upbeta }$) of main effects of gender, main effects of age, and interaction effects

*ref* Reference group

We also wanted to analyze the likelihood that one would misperceive oneself as being underweight. Comparing male participants only, we found no significant difference in the risk of misperception of being underweight among the three age groups (*p *= 0.39, *p *= 0.92). Comparing men and women, we found no significant difference in perceiving underweight between the older males and females (*p *= 0.16). However, younger and middle-aged women were less likely to misperceive themselves as being underweight than their younger and middle-aged male counterparts (OR = 0.15; OR = 0.32 respectively).

Different cutoff points for BMI may produce different results. The Asia–Pacific classification of BMI has lower cutoffs for overweight and obese categories than the WHO classification [29]. For comparison, BMI was also categorized into three groups according to the Asia–Pacific classification: underweight (< 18.5 kg/m^2)^, normal weight (18.5–22.9 kg/m^2^), and overweight (≥ 23 kg/m^2^). Results showed that percentages in the underweight category for both WHO (5.7%) and Asia standards are almost identical (5.8%) for the whole population. That percentage in the normal weight category was lower using Asia standard than that using WHO standard (WHO: 61.1% vs. Asia: 41.4%); percentages in the overweight category was higher using Asian standard than that using WHO standard (WHO: 33.2% vs. Asia: 52.8%). For concordance between measured and self-perceived weight, WHO standard showed a lower percentage than Asia standard for misperceived underweight (WHO: 15.3% vs. Asia: 23.2%). WHO standard showed a higher percentage than Asia standard for misperceived overweight (WHO: 23.4% vs. Asia: 12.6%) (Additional file 1: Appendix A). Based on misperceived overweight and underweight using Asian standard, nominal logistic regression models showed that the directions for all estimated parameters in the two results were the same (Additional file 1: Appendices B and C).

We also aimed to analyze the likelihood of individuals misperceiving themselves as underweight. For both WHO and Asian standards, younger and middle-aged women were less likely to misperceive themselves as underweight than their younger and middle-aged male counterparts (WHO standards: OR = 0.15; OR = 0.32, respectively; Asian standards: OR = 0.08; OR = 0.20, respectively). Among male participants only, we found no significant difference in the risk of misperceiving underweight across the three age groups using WHO standards (*p *= 0.39, *p *= 0.92). However, when using Asian standards, we discovered that young males were less likely than older males to misperceive themselves as underweight (*p *= 0.024).

## Discussion

Women are commonly thought to care more about their weight or body size than men. Based on this common belief and the stereotypes perpetuated by popular culture and the media, it may be understandable that we maintain the opinion that women are more likely to misperceive their weight and body size [30]. However, there are many instances in which science has proved popular thought to be incorrect. Our study sought to identify differences in weight misperception in healthy adult males and females of Taiwanese population in different age groups. Most studies of weight perception have been performed in the West among young females or women with eating disorders who misperceive themselves as being too big [31]. The findings of our population-based study underscore a significant interaction effect between sex, age, and their consequent influence on weight misperception. Specifically, we observed that Taiwanese women were more likely to misperceive themselves as overweight, while men were more likely to perceive themselves as underweight. This sex-based disparity in weight misperception was found to be significantly influenced by age. However, it's important to note that this interaction effect diminished in the older age groups, indicating that the influence of sex and age on weight misperception becomes less pronounced as individuals age.

Different cutoff points for BMI may produce different results. Underweight percentages are similar for WHO and Asia standards, while normal weight decreases and overweight increases in Asia standards. Asia standards show higher misperceived underweight and lower misperceived overweight percentages than WHO standards. Women of all ages were more likely to perceive themselves as overweight than men, with higher ratios in Asian standards. Younger and middle-aged women were less likely to misperceive themselves as underweight compared to men in both standards.

### WHO BMI standards versus Asia BMI standards

Different BMI standards may affect multiple aspects of public health and individual well-being. First, regarding public health policy and awareness, different BMI standards can influence policies, guidelines, and resource allocation, as well as the reporting of overweight or obesity prevalence. Our study revealed that Asia standards (52.8%) for overweight cases were considerably higher than WHO standards (33.2%). These varying criteria can steer policy and resource allocation in different directions. Secondly, in clinical practice, healthcare providers may need to adopt different BMI standards for diagnosis and treatment, depending on the cultural and regional context, potentially leading to inconsistencies in patient care. Thirdly, at the individual level, different BMI standards may affect people's perceptions of their body weight, influencing self-esteem, body image, and mental well-being. Lastly, research on BMI-health outcome associations may yield varying results based on the standards used, affecting the generalizability and applicability of the findings.

### Weight misperception and overweight prevalence

The four waves of Taiwan's National Nutrition and Health Status Change Survey (1993–1996, 2005–2008, 2013–2016, and 2017–2020) demonstrate a decline in normal-weight individuals from 58.1% to 45.0%, an increase in overweight and obese individuals from 33.2% to 50.6%, and a rise in morbid obesity prevalence from 0.44% to 1.56% [32]. In 2016, 39% of global adults were overweight or obese (BMI ≥ 25) [33]. The misperception of being underweight may be one of the determinant factors leading to obesity since individuals who perceive themselves as underweight may be less likely to exercise or impose dietary restrictions, ultimately contributing to weight gain. Further research is needed to explore the potential effects of the discrepancy between actual and self-perceived weight.

### Factors related to weight perception

Previous studies [11, 16, 34] have found that individuals with higher BMI, females, those with higher income, white individuals, and those with higher education are more likely to perceive themselves as overweight. Our study aligns with these findings, as we discovered that females, younger individuals, and those with lower perceived social status are more likely to perceive themselves as overweight. Consistent with previous studies [15, 34, 35], our research findings indicated that weight misperception of being overweight highly correlates with females and individuals with higher educational levels. The mechanisms or causal factors influencing the perception of being overweight and weight misperception may be affected by various factors, such as cognitive, social, and individual aspects. For example, media exposure and social comparison can contribute to body dissatisfaction and disordered eating patterns by promoting unrealistic beauty standards [36, 37]. Social comparison contributes to body dissatisfaction and disordered eating patterns as individuals internalize and compare themselves to unrealistic beauty standards [38, 39]. One possible explanation for this finding is that older adults, who use social media less, may be less prone to compare themselves to content on social media socially, thus reducing the likelihood of experiencing body image disturbance [37]. Another possible mechanism is that older individuals tend to engage in protective social comparisons (e.g., comparing themselves to targets perceived as inferior), which may mitigate the adverse effects of social media use [40].

### Culture and body size perception

This study found that 58.4% (Asia standards: 64.2%) of the subjects in our population-based study had an accurate perception of their weight, similar to a proportion reported in a previous Korean study (58.5%) [16]. This proportion was much lower than that reported by two studies in the United States, 73.9% and 71.4% [14, 35], and it was much higher than the 41.5% reported by a study in Nigeria [17]. Our study shows that misperceived underweight is more common among older adults (WHO: 25.2%, Asia: 39.7%) than younger ones (WHO: 10.5%, Asia: 23.2%), possibly due to adherence to traditional beliefs. In Taiwan, the preference for fatness may be rooted in historical beauty ideals, such as the fuller-sized women of the Tang Dynasty (618–907 CE) represented by "Tang Beauties" sculptures [41]. Societal urbanization and industrialization influenced beauty standards, associating thinner bodies with higher social status [42], while Westernization and modernization shifted global ideals towards slimmer body types [30]. Contrary to Western culture, which associates thinness with attractiveness and success [43]. Chinese culture historically considered fatness as an emblem of beauty and wealth [44], Although Asian societies, like their Western counterparts, have witnessed a shift in preference towards slimmer body types, this trend seems to be more pronounced among the younger generation [45, 46]. Therefore, as observed in our study agrees with the prior findings on Chinese youths, which showed that Chinese youths intend to overestimate their body sizes because they prefer slimming shapes. Health issues highlight the greater comorbidity risks involved with being overweight, specifically for older adults, demanding an emphasis on health-centered weight management over traditional perspectives. Individuals with overweight are at an increased risk of cardiovascular diseases, diabetes, and some physical cancers [33]. Therefore, public health campaigns should be tailored to older adults, endorsing balanced diets and routine physical activity to keep a healthy weight and lessen chronic disease risks, while considering the weight myth linked to prosperity and quality living for the elderly.

### Age and body size perception

This study found a much greater percentage of younger and middle-aged people than older people misperceived themselves as being overweight (WHO: 31.5%, and 20.7% vs. 11.1%; Asia: 21.2% and 8.3%, and 3.7% respectively). The trend was reversed for misperceptions of being too thin (WHO: 10.5%, 16.0%, and 25.2%; Asia: 15.8%, 23.9%, and 39.7%, respectively), which is important information for a field that lacks data in this area. Similarly, Joo et al. [34] analyzed data from 22,121 women (aged 19–97 years) in the 7-year Korea National Health and Nutrition Examination Survey dataset (2010–2016), using Asian standards . They also found that overweight misperception decreased from 14.1% for young adults to 2.2% for older female adults, while underweight misperception increased from 3.4% for younger adults (aged 19–45) to 29.6% for older adults (aged 60 and above).

As individuals age, their priorities often shift, leading older people to value health more than body image [47]. Older adults may also appreciate their body's functionality more than its appearance, helping protect them from body dissatisfaction and disordered eating more common in younger populations [48]. This focus on health and well-being over body image concerns is reinforced by research showing women and men prioritizing competence and abilities over appearance as they age [49]. Furthermore, older individuals are more likely to engage in protective social comparisons (e.g., comparing themselves to less fit or less healthy individuals), which may help maintain self-esteem and reduce the impact of social media's unrealistic body ideals [40]. Addressing body image concerns in healthy aging policies and practices is essential, as body dissatisfaction remains consistent across age groups.

### Gender and misperception of weight

This study found women, especially younger women, more likely than men to misperceive themselves as being overweight while men, particularly younger men, were more likely than women to misperceive themselves as being too thin. The reason that younger women have this misperception has been well-studied, especially among those with eating disorders. More research is needed to study male misperceptions of being too thin, perhaps research investigating whether images of strength, health, or vitality young men may want to project have an effect on this misperception [50]. Although recent studies have found the prevalence of eating disorders to be increasing among men [51], no research has focused on changes in self-perception of weight among healthy males. Interestingly, this study found that older men and women were more likely to misperceive themselves as being too thin (25.2%). The reason that women may be concerned with possibly being underweight may be cosmetic in nature. With aging, comes a loss of under-skin collagen, loss of skin elasticity, and wrinkling. The loss of facial fat may exacerbate the visual signs of aging. Thus, some women, as well as some men, may look at the aging skin of their faces and misperceive themselves as being underweight and looking old. This possibility might explain the reason that, fat grafting of small amounts of the patient’s own fat to strategic areas of the face has become a common procedure practiced by cosmetic surgeons [52]. In addition, in the minds of older people, thinness may be closely associated with fragility and vulnerability to physical dysfunction. These two fears, aging and fragility, may lead older people to be less concerned about losing weight and more interested in maintaining or increasing bulk by eating more, which depending upon their dietary and lifestyle habits could be healthy or not.

### Limitations

This study is stronger than most weight perception studies in that it has a more complete database and has included a wider variety of relevant variables. However, it has several limitations. One of the most important limitations is that the analyzed data were collected in 2011; therefore, the results should be interpreted cautiously. The perspectives on weight image and concerns in the present study might differ from the current era. Nevertheless, the present findings could be useful for future studies to take the reference as an early-stage conceptual model [53, 54]. We had BMI calculations based on self-reported height and weight, and it is possible that some of the subjects overestimated their height and underestimated their weight, which could have had a small effect on our calculations. However, some studies found a strong correlation between measured and self-reported weight (r = 0.82–0.99, *p *< 0.001) [55, 56]. These findings demonstrate substantial to a near-perfect agreement in weight classification [55, 56]. Still another limitation is the way we interviewed subjects about their perceptions of body size. We asked the subjects how they viewed themselves (underweight, slightly underweight, neither underweight nor overweight, slightly overweight, and overweight), another way of asking could be to show subjects pictures of various body sizes and ask them to match the picture closest to their perception of their own shape. This might have produced different results, though further studies comparing the two ways might be performed to confirm this.

There are many directions that future study can take. Firstly, there is a noticeable gap in research concerning weight perception as compared to weight misperception. The factors affecting these two aspects are inversely different, which may be attributed to the complex interplay of cultural, policy, and individual levels. Furthermore, our findings agree with other Asian research [34], showing that people misperceive themselves as being underweight rather than overweight. This can lead to an increase in the number of overweight individuals who may not be properly managing their weight, particularly among the older cohort. Further research is needed to investigate the factors contributing to the misperception of being underweight and its potential impacts on weight gain, including aspects such as quality of life, physical health, and daily social activities. Secondly, weight management is a lifelong concern, especially for women. As individuals age and confront the reality of growing older, prioritizing competency and health over physical appearance may become an essential factor contributing to life quality in late adulthood. This highlights the need for more research on positive aging and its implications for overall well-being.

## Conclusions

In conclusion, this study found that age and gender had a clear effect on self-perceptions of body size in Taiwan, with women generally more likely to misperceive themselves as being too big and men more likely to misperceive themselves as being too thin. Older people were less concerned with their body shape than younger people, possibly because body size may hold different meanings for them. The preliminary findings of this relatively new area of study, misperception, may be used by education and exercise programs. Clinicians and trainers may need to be reminded that the weight concerns of people vary by gender as well as age.

## Supplementary Information

**Additional file 1**. **Appendix A**. Characteristics of subjects by age group for Asia standards. **Appendix B**. Nominal logistic regression models analyzing select subject characteristics by misperceived overweight and misperceived underweight. **Appendix C**. Main effects, interaction effects, and the total effect of gender and age on the misperception of overweight and underweight.

## Data Availability

Ying-hwa Chang (2016). 2011 Taiwan Social Change Survey (Round 6, Year 2): Health (C00222_2) [20]. Available from Survey Research Data Archive, Academia Sinica. https://doi.org/10.6141/TW-SRDA-C00222_2-1
