# Supplementary material for: Age and gender differences in misperceptions of body shape in a Taiwanese population
Source: J Eat Disord. 2023 Jul 3;11:110. doi: 10.1186/s40337-023-00837-5 (PMC10318745; doi:10.1186/s40337-023-00837-5)
Supplement: Supplementary file 1 — Additional file 1. Appendix A. Characteristics of subjects by age group for Asia standards. Appendix B. Nominal logistic regression models analyzing select subject characteristics by misperceived overweight and misperceived underweight. Appendix C. Main effects, interaction effects, and the total effect of gender and age on the misperception of overweight and underweight. [file 40337_2023_837_MOESM1_ESM.docx]

Supplementary Materials

Appendix A Characteristics of subjects by age group for Asia standards

| Variable | Age groups | | | | | | | |  |
| --- | --- | --- | --- | --- | --- | --- | --- | --- | --- |
|  | All | | 18-35 | | 36-64 | | ≥ 65 | |  |
|  | (n=2199) | | (n=838) | | (n=977) | | (n=384) | | p |
|  | mean | SD | Mean | SD | mean | SD | mean | SD |  |
| BMI (n=2095) | 23.6 | 3.8 | 22.8 | 4.2 | 24.3 | 3.4 | 23.9 | 3.3 | <0.001 |
| Perceived household income (1-5) | 2.7 | 0.7 | 2.8 | 0.6 | 2.7 | 0.7 | 2.6 | 0.8 | <0.001 |
| Perceived social status (1-10) | 5.0 | 1.6 | 5.0 | 1.5 | 4.9 | 1.6 | 4.9 | 1.7 | 0.225 |
|  | n | % | N | % | n | % | n | % | p |
| Gender (female) | 1113 | 50.6 | 402 | 48.0 | 513 | 52.5 | 198 | 51.6 | 0.144 |
| Marital status (married) | 1299 | 59.1 | 256 | 30.5 | 790 | 80.9 | 253 | 65.9 | <0.001 |
| Job (with job) | 1370 | 62.3 | 642 | 76.6 | 667 | 68.3 | 61 | 15.9 | <0.001 |
| Educational level | | | | | | | | |  |
| Primary school and below | 475 | 21.6 | 2 | 0.2 | 222 | 22.7 | 251 | 65.4 | <0.001 |
| High school | 816 | 37.1 | 247 | 29.5 | 482 | 49.3 | 87 | 22.7 | <0.001 |
| College and above | 908 | 41.3 | 589 | 70.3 | 273 | 27.9 | 46 | 12.0 | <0.001 |
| BMI group | | | | | | | | | <0.001 |
| BMI≤18.5 (Underweight) | 119 | 5.7 | 87 | 10.6 | 21 | 2.2 | 11 | 3.4 |  |
| BMI 18.5-22.9 (Normal weight) | 869 | 41.4 | 400 | 48.7 | 349 | 36.7 | 120 | 36.9 |  |
| BMI≥23 (Overweight) | 1107 | 52.8 | 335 | 40.8 | 578 | 61.0 | 194 | 59.7 |  |
| Weight perception | | | | | | | | | <0.001 |
| Underweight | 299 | 13.6 | 136 | 16.2 | 106 | 10.8 | 57 | 14.8 |  |
| Normal | 822 | 37.4 | 263 | 31.4 | 351 | 35.9 | 208 | 54.2 |  |
| Overweight | 1078 | 49.0 | 439 | 52.4 | 520 | 53.2 | 119 | 31.0 |  |
| Concordance between measured weight and self-weight perception | | | | | | | | | <0.001 |
| Misperceived underweight | 486 | 23.2 | 130 | 15.8 | 227 | 23.9 | 129 | 39.7 |  |
| Rightly perceived weight | 1344 | 64.2 | 518 | 63.0 | 642 | 67.7 | 184 | 56.6 |  |
| Misperceived overweight | 265 | 12.6 | 174 | 21.2 | 79 | 8.3 | 12 | 3.7 |  |

Appendix B Nominal logistic regression models analyzing select subject characteristics by misperceived overweight and misperceived underweight

|  | Misperceived Overweight | | | | |
| --- | --- | --- | --- | --- | --- |
|  | Coefficient (β) | p | OR | 95% CI | |
| Variables |  |  |  | Lower | Upper |
| Intercept | -3.1 | <0.001 | -- | -- | -- |
| Young age | 0.35 | 0.585 | 1.41 | 0.41 | 4.88 |
| Middle age | -1.2 | 0.123 | 0.30 | 0.01 | 1.38 |
| Gender (male=ref) | 1.52 | 0.029 | 4.59 | 1.17 | 18.01 |
| Educational level (elementary school or below = ref) | | | |  |  |
| high school | 1.26 | <0.001 | 3.53 | 1.79 | 6.98 |
| college or above | 1.68 | <0.001 | 5.34 | 2.64 | 10.83 |
| Perceived social status (1-10) | -0.11 | 0.003 | 0.90 | 0.81 | 0.99 |
| Young age*female | 0.62 | 0.400 | 1.86 | 0.44 | 7.92 |
| Middle age*female | 1.43 | 0.100 | 4.17 | 0.76 | 22.85 |
|  | Misperceived Underweight | | | | |
| Variables | Coefficient (β) | p | OR | 95% CI | |
|  |  |  |  | Lower | Upper |
| Intercept | 0.29 | 0.235 | -- | -- | -- |
| Young age | -0.47 | 0.024 | 0.62 | 0.42 | 0.94 |
| Middle age | -0.29 | 0.122 | 0.75 | 0.51 | 1.08 |
| Gender (male=ref) | -0.87 | <0.001 | 0.42 | 0.26 | 0.67 |
| Educational level (elementary school or below = ref) | | | |  |  |
| high school | -0.55 | 0.001 | 0.58 | 0.42 | 0.79 |
| college or above | -0.77 | <0.001 | 0.46 | 0.32 | 0.67 |
| Perceived social status (1-10) | -0.00 | 0.921 | 1.00 | 0.93 | 1.07 |
| Young age*female | -1.19 | 0.003 | 0.31 | 0.14 | 0.67 |
| Middle age*female | -0.47 | 0.110 | 0.63 | 0.35 | 1.11 |

Appendix C Main effects, interaction effects, and the total effect of gender and age on the misperception of overweight and underweight

| Main effects of Gender | | Main effects of Age | | Interaction Effects | Total Effects | | | |
| --- | --- | --- | --- | --- | --- | --- | --- | --- |
| Misperceived overweight | Coefficient ($\hat{\beta}$) |  | Coefficient ($\hat{\beta}$) | Coefficient ($\hat{\beta}$) | Coefficient ($\hat{\beta}$) | s.e. | p | OR |
| Female | 1.52 | Young | 0.35 | 0.62 | 2.49 | 0.60 | <0.001 | 12.07 |
|  |  | Middle | -1.20 | 1.43 | 1.76 | 0.60 | 0.004 | 5.78 |
|  |  | Old (ref) |  |  | 1.52 | 0.70 | 0.029 | 4.59 |
| Male (ref) |  | Young | 0.35 |  | 0.35 | 0.63 | 0.585 | 1.41 |
|  |  | Middle | -1.20 |  | -1.20 | 0.78 | 0.123 | 0.30 |
|  |  | Old (ref) |  |  |  |  | | |
|  |  |  |  |  |  |  |  |  |
| Misperceived underweight | Coefficient ($\hat{\beta}$) |  | Coefficient ($\hat{\beta}$) | Coefficient ($\hat{\beta}$) | Coefficient ($\hat{\beta}$) | s.e. | p | OR |
| Female | -0.87 | Young | -0.47 | -1.19 | -2.53 | 0.35 | <0.001 | 0.08 |
|  |  | Middle | -0.29 | -0.47 | -1.64 | 0.21 | <0.001 | 0.20 |
|  |  | Old (ref) |  |  | -0.87 | 0.24 | <0.001 | 0.42 |
| Male (ref) |  | Young | -0.47 |  | -0.47 | 0.21 | 0.024 | 0.62 |
|  |  | Middle | -0.29 |  | -0.30 | 0.19 | 0.122 | 0.75 |
|  |  | Old (ref) |  |  |  |  | | |

Note: Total effects are the sum of Coefficient (β) of main effects of gender, main effects of age, and interaction effects.
